# Supplementary material for: Asynchronous magnetic resonance elastography: Shear wave speed reconstruction using noise correlation of incoherent waves
Source: Magn Reson Med. 2022 Oct 27;89(3):990–1001. doi: 10.1002/mrm.29502 (PMC9792433; doi:10.1002/mrm.29502)
Supplement: Supplementary file 1 — DATA S1 MATLAB code used to generate the numerical simulations of Figure 1 [file MRM-89-990-s002.zip › k-Wave/helpfiles/example_diff_homogeneous_medium_source.html]

Constant Rate Of Heat Deposition (k-Wave)


# Constant Rate Of Heat Deposition

This example provides a simple demonstration of using k-Wave to model the diffusion of heat within a two-dimensional homogeneous medium with a constant source term (volume rate of heat deposition). It builds on the Heat Diffusion In A Homogeneous Medium example.

- Open the file in the MATLAB Editor
- Run the file in MATLAB

## Contents

- Defining the medium properties
- Defining the heat source term
- Calculating thermal dose
- Comparison with exact solution

## Defining the medium properties

In the Heat Diffusion In A Homogeneous Medium Example, only the parameters related to diffusion are defined. Here, the parameters related to tissue perfusion are also specified. Similar to the diffusion parameters, these can be defined in two ways, either by defining a single perfusion coefficient (assigned to `medium.perfusion_coeff`), or by defining the density, specific heat capacity, and perfusion rate of the blood (assigned to `medium.blood_density`, `medium.blood_specific_heat`, and `medium.blood_perfusion_rate`). In both cases, the ambient temperature of the blood must be defined (assigned to `medium.blood_ambient_temperature`). These parameters can be specified as single scalar values in SI units (for homogeneous coefficients), or as matrices the same size as the computational grid (for heterogeneous coefficients). In this example, the individual medium properties are set as scalar values.

```
% define medium properties related to diffusion
medium.density                      = 1079;     % [kg/m^3]
medium.thermal_conductivity         = 0.52;     % [W/(m.K)]
medium.specific_heat                = 3540;     % [J/(kg.K)]

% define medium properties related to perfusion
medium.blood_density                = 1060;     % [kg/m^3]
medium.blood_specific_heat          = 3617;     % [J/(kg.K)]
medium.blood_perfusion_rate         = 0.01;     % [1/s]
medium.blood_ambient_temperature    = 37;       % [degC]
```

## Defining the heat source term

In this example, the initial temperature is set to body temperature, and a volume heat source term (defined in units of W/m^3) is set to be a Gaussian distribution. The simulation is then performed by calling the `takeTimeStep` method.

```
% set initial temperature distribution to be constant [degC]
source.T0 = 37;

% set Gaussian volume rate of heat deposition [W/m^3]
width = 4*dx;
source.Q = 2e6 .* exp( -(kgrid.x ./ width).^2 - (kgrid.y ./ width).^2 );
```

## Calculating thermal dose

During the simulation, the thermal dose delivered to the tissue is automatically calculated in units of cumulative equivalent minutes. This is calculated using the formula `cem43 = cem43 + dt/60 * R ^(43 - T)`, where `dt` is the size of the time step in seconds. The parameter `R` is given by 0.5 if the temperature `T` is above 43 degrees Celsius, and 0.25 if it is below (the difference in slope below 43 is due to the development of thermo-tolerance during heating). The dependence on `dt` means that if the temperature is rapidly changing, using a smaller time step will give a more accurate estimate of the thermal dose. The thermal dose can be accessed from an object of the `kWaveDiffusion` class using the dynamic property `kdiff.cem43`. A thresholded lesion map where `cem43` exceeds 240 cumulative equivalent minutes can also be returned using the property `kdiff.lesion_map`. These are plotted below. The total size of the lesion can also be returned using the property `kdiff.lesion_size`. This is given in units of distance in 1D [m], area in 2D [m^2], and volume in 3D [m^3].


## Comparison with exact solution

For a constant source term, when the medium is homogeneous, the computation by `kWaveDiffusion` is again exact and unconditionally stable. In this case, the calculations using `kWaveDiffusion` and `bioheatExact` agree to machine precision.

```
% calculate perfusion coefficient from the medium parameters
P = medium.blood_density .* medium.blood_perfusion_rate .* ...
    medium.blood_specific_heat ./ (medium.density .* medium.specific_heat);

% calculate diffusivity from the medium parameters
D = medium.thermal_conductivity / (medium.density * medium.specific_heat);

% calculate normalised heat source
S = source.Q ./ (medium.density .* medium.specific_heat);

% compute Green's function solution using bioheatExact
T_exact = bioheatExact(source.T0 .* ones(Nx, Ny), S, ...
    [D, P, medium.blood_ambient_temperature], kgrid.dx, Nt * dt);
```
